# Aspirin is associated with improved outcomes in sepsis patients with atrial fibrillation: an analysis of the MIMIC-IV and eICU-CRD databases

**DOI:** 10.3389/fcvm.2026.1796096

**Published:** 2026-05-26

**Authors:** Fangchao Chen, Yufeng Zhong, Dianyang Wang, Qiuyin Wei, Rui Su, Hongfei Ge, Wencai Wei, Wei Wang

**Affiliations:** 1 Department of Emergency, The First Affiliated Hospital of Guangxi Medical University, Nanning, China; 2 Department of Emergency, Liuzhou Workers' Hospital, The Fourth Affiliated Hospital of Guangxi Medical University, Liuzhou, China; 3 Guangxi University Key Laboratory of Emergency Medicine, The First Affiliated Hospital of Guangxi Medical University, Nanning, China; 4 Department of Respiratory and Critical Care Medicine, Liuzhou Workers' Hospital, The Fourth Affiliated Hospital of Guangxi Medical University, Liuzhou, China; 5 Department of Critical Care Medicine, Liuzhou Workers' Hospital, The Fourth Affiliated Hospital of Guangxi Medical University, Liuzhou, China; 6 Department of Cardiology, Liuzhou Workers' Hospital, The Fourth Affiliated Hospital of Guangxi Medical University, Liuzhou, China

**Keywords:** aspirin, atrial fibrillation, clinical outcomes, MIMIC-IV, sepsis

## Abstract

**Background:**

Sepsis is characterized by dysregulated inflammation, endothelial injury, platelet activation, and immunothrombosis. In patients with atrial fibrillation (AF), these processes may further increase thrombotic risk and worsen clinical outcomes. Aspirin may be relevant in this setting because of its antiplatelet and anti-inflammatory properties; however, evidence in sepsis patients with AF remains limited, and exposure timing complicates interpretation.

**Objective:**

To evaluate the association between early aspirin exposure and mortality in sepsis patients with AF.

**Methods:**

We conducted a retrospective cohort study using MIMIC-IV with external validation in eICU-CRD. Patients were classified according to aspirin exposure before ICU admission or within 48 h after ICU admission vs. no aspirin exposure within 48 h. A three-category sensitivity analysis further separated pre-ICU aspirin exposure from newly initiated aspirin within 48 h after ICU admission. In MIMIC-IV, the primary outcome was 30-day all-cause mortality and the secondary outcome was in-hospital mortality. Validation outcomes were in-hospital mortality and 30-day in-hospital mortality. Kaplan–Meier analysis, multivariable Cox regression, subgroup analyses, and weighted sensitivity analyses were performed.

**Results:**

In MIMIC-IV, 8,827 patients with sepsis and AF were included, of whom 2,175 had early aspirin exposure. Early aspirin exposure was associated with lower 30-day all-cause mortality; in the fully adjusted model, the hazard ratio was 0.624 (95% CI: 0.547–0.712; *P* < 0.001). In overlap-weighted analysis, this association remained significant (HR: 0.738, 95% CI: 0.647–0.842; *P* < 0.001). In the three-category sensitivity analysis, the inverse association was observed mainly among patients newly initiated on aspirin within 48 h after ICU admission. In eICU-CRD, early aspirin exposure was associated with lower in-hospital mortality in the fully adjusted model (HR: 0.732, 95% CI: 0.559–0.959; *P* = 0.024), whereas the association with 30-day in-hospital mortality was attenuated.

**Conclusion:**

Early aspirin exposure was associated with lower 30-day mortality in sepsis patients with AF, particularly among patients newly started on aspirin during early ICU care; associations with in-hospital mortality were less consistent.

## 1 Introduction

Sepsis is a dysregulated host response to infection that results in life-threatening organ dysfunction. Its pathophysiology involves systemic inflammation, immune dysregulation, endothelial injury, and activation of the coagulation cascade, all of which contribute to microcirculatory dysfunction and multiple organ failure (1). Cardiovascular complications are common in sepsis and are closely associated with poor prognosis (2). Among these complications, atrial fibrillation (AF) is of particular importance because it may arise in response to inflammation, autonomic imbalance, myocardial depression, endotoxemia, and underlying structural heart disease (3, 4). AF can further impair haemodynamic stability, reduce cardiac output, and aggravate tissue hypoperfusion, thereby worsening organ dysfunction in critically ill patients (5–7).

Aspirin is a classic cyclooxygenase inhibitor with antiplatelet, anti-inflammatory, and potentially immunomodulatory properties (8–11). By suppressing thromboxane synthesis and modulating inflammatory signalling, aspirin may influence the interplay between thrombosis and inflammation that characterizes sepsis. This biological plausibility is supported by the platelet–inflammation–thrombosis axis, in which activated platelets express CD40L and CD40/CD40L signalling links platelet activation to inflammatory and thrombotic responses (12). Previous studies have suggested that antiplatelet therapy may be associated with improved outcomes in selected sepsis populations (13, 14),although the findings have been inconsistent.Some reports have further indicated that antiplatelet therapy, whether initiated before or after sepsis onset, may reduce mortality by 22%–66% (15–17), whereas others have found no clear benefit (18, 19). Nevertheless, evidence supports potential organ-protective effects of aspirin in specific sepsis phenotypes, including acute respiratory distress syndrome (ARDS) (20, 21), acute kidney injury (AKI) (22), and myocardial injury (23). In addition, aspirin has been studied in broader critically ill AF populations, but evidence specifically focused on sepsis patients with AF remains limited (4).

This clinical context is particularly complex because AF management usually centers on anticoagulation-based stroke prevention, and the role of aspirin in AF has become increasingly restricted in routine cardiovascular care (24–27). Nevertheless, sepsis-associated AF represents a distinct inflammatory and prothrombotic state, and whether aspirin may still have prognostic relevance in this phenotype remains uncertain. Importantly, prior observational studies are vulnerable to time-related bias if aspirin is treated simply as an in-hospital exposure without adequate consideration of when treatment was actually initiated.

Accordingly, the present study aimed to evaluate the association between early aspirin exposure and mortality in sepsis patients with AF using a 48-hour exposure window after ICU admission. We used MIMIC-IV as the primary cohort, performed external validation in eICU-CRD, and further distinguished aspirin exposure before ICU admission from newly initiated aspirin within 48 h after ICU admission in sensitivity analyses.

## 2 Materials and methods

### 2.1 Data sources and ethical review

Data were obtained from the publicly available MIMIC-IV database (version 3.1, PhysioNet) (28). This database has been approved by the Institutional Review Boards (IRB) of Beth Israel Deaconess Medical Center (BIDMC) and the Massachusetts Institute of Technology (MIT) (29). External validation data were derived from the eICU Collaborative Research Database (eICU-CRD) (version 2.0) (30), which contains de-identified records from more than 200,000 ICU admissions across over 200 hospitals in the United States between 2014 and 2015. The principal investigator completed the Collaborative Institutional Training Initiative (CITI) program (Certification No. 72379798) and signed the data use agreement to access both databases. Because this study was a retrospective analysis of de-identified data, the requirement for informed consent was waived by the IRB.

### 2.2 Study population

Adult patients with sepsis and AF were identified in the MIMIC-IV and eICU-CRD databases. The inclusion criteria were as follows: (1) age ≥18 years; (2) ICU admission meeting Sepsis-3 criteria, defined as suspected or confirmed infection with an acute increase in Sequential Organ Failure Assessment (SOFA) score ≥2; (3) AF included patients with a history of AF and those with diagnosed AF documented before ICU admission; (4) availability of key study variables, including vital signs and laboratory values; (5) for patients with multiple ICU admissions, only the first admission was included; and (6) in the main analysis, patients were classified as having early aspirin exposure if they had aspirin exposure before ICU admission or within 48 h after ICU admission, and as non-aspirin if no aspirin exposure occurred within 48 h after ICU admission. In sensitivity analyses, patients were further classified into three groups: non-aspirin within 48 h, aspirin exposure before ICU admission, and newly initiated aspirin within 48 h after ICU admission. The exclusion criteria were: (1) ICU length of stay <48 h; and (2) missing data exceeding 20% of the total dataset in MIMIC-IV or 30% in eICU-CRD.

### 2.3 Data collection

Baseline data included: (1) aspirin prescription information, including use status, duration, cumulative dose, and timing relative to sepsis; (2) demographic characteristics, including age, sex, ethnicity, and weight; (3) severity scores at admission, including SOFA, GCS, and APACHE II; (4) comorbidities, including hypertension (HTN), valvular heart disease (VHD), ischemic stroke (IS), diabetes mellitus (DM), chronic kidney disease (CKD), cancer, myocardial infarction (MI), coronary artery disease (CAD), acute kidney injury (AKI), hyperlipidemia (HLD), and heart failure (HF); (5) initial vital signs, including HR, RR, SBP, DBP, temperature, and SpO₂; (6) initial laboratory measurements, including white blood cell count (WBC), hemoglobin (Hb), platelet count (PLT), anion gap (AG), glucose (Glu), potassium (K), sodium (Na), total calcium (Ca), pH, arterial partial pressure of oxygen (PaO₂), lactate (Lac), international normalized ratio (INR), prothrombin time (PT), serum creatinine (Cr), and blood urea nitrogen (BUN); and (7) initial interventions, including warfarin, beta-blockers, non-vitamin K antagonist oral anticoagulants (NOACs), continuous renal replacement therapy (CRRT), and invasive mechanical ventilation (MV). Data were extracted using SQL with PostgreSQL 15.0 from MIMIC-IV (v3.1) and eICU-CRD (v2.0). All baseline variables were collected within 24 h of ICU admission.

### 2.4 Exposure and outcomes

The exposure of interest was aspirin exposure before ICU admission or within 48 h after ICU admission. In MIMIC-IV, the primary outcome was 30-day all-cause mortality and the secondary outcome was in-hospital mortality. Because eICU-CRD does not provide comparable long-term post-discharge follow-up, the external validation outcomes were in-hospital mortality and 30-day in-hospital mortality.

### 2.5 Statistical analysis

Continuous variables are presented as medians with interquartile ranges, and categorical variables are presented as counts with percentages. Baseline characteristics were compared between exposure groups. Kaplan–Meier curves and log-rank tests were used to compare survival. Cox proportional hazards models were constructed sequentially. Model 1 was unadjusted. Model 2 adjusted for demographic characteristics, selected admission vital signs, comorbidities, and SOFA score. Model 3 further adjusted for selected laboratory variables and treatment variables, as specified in the table notes of the final results. To further examine the robustness of the findings, overlap-weighted analyses anchored to the 48-hour exposure framework were also performed. Subgroup analyses were conducted for 30-day mortality. Missingness of baseline variables in both databases was summarized in the Supplementary Material. This study followed the RECORD statement (31).

## 3 Results

### 3.1 Baseline characteristics in the primary cohort

**Figure 1:**
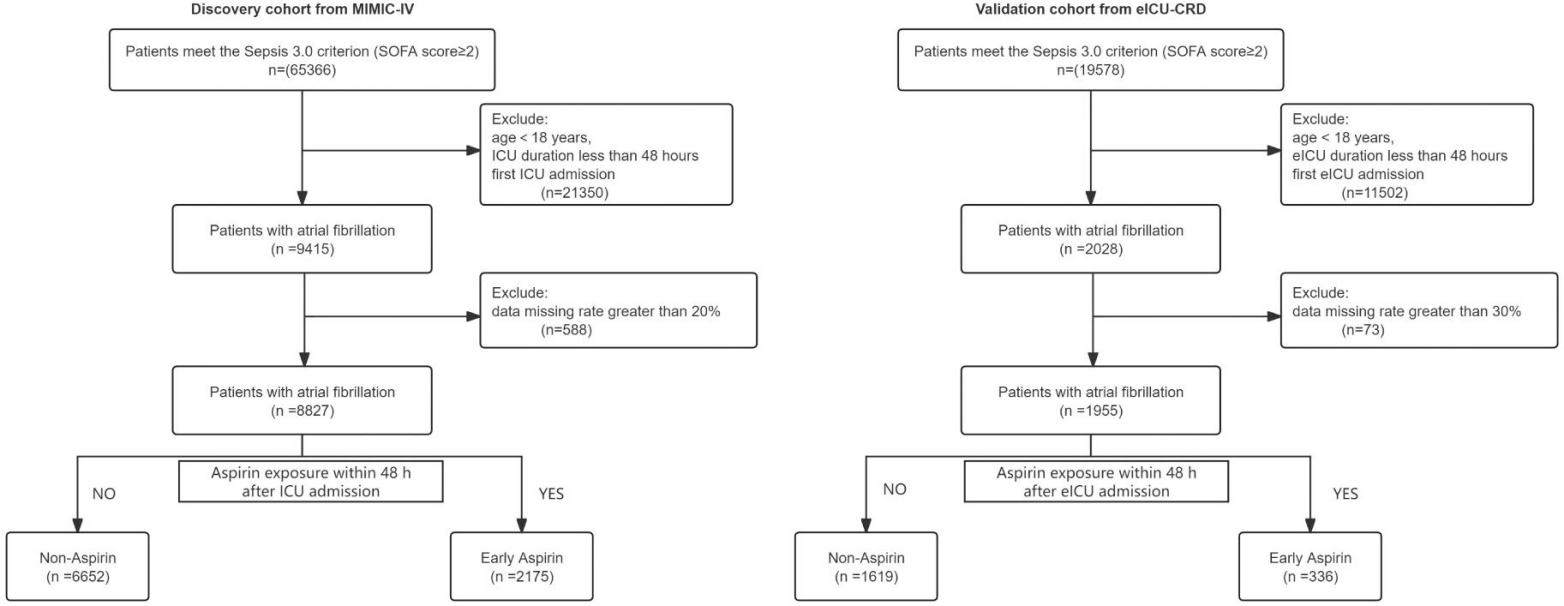
Flowchart of the study.

In the MIMIC-IV cohort, 8,827 patients with sepsis and AF were included in the analysis, comprising 6,652 patients without aspirin exposure within 48 h after ICU admission and 2,175 patients with early aspirin exposure (Figure 1). Compared with the non-aspirin group, patients in the early aspirin group were younger, more often male, and had slightly lower SOFA and APACHE II scores. Early aspirin use was more frequent among patients with VHD, HTN, HLD, and CAD, whereas no significant differences were observed for IS or MI. The early aspirin group had lower rates of CRRT but higher rates of MV. Medication use also differed between groups: beta-blocker use was more frequent in the early aspirin group, whereas NOAC use was less frequent; warfarin use did not differ significantly between groups (Table 1).

**Table 1:** Baseline characteristics of sepsis patients with AF according to early aspirin exposure in the MIMIC-IV database.

Variable	Overall	Non- aspirin	Early aspirin	*P* value	SMD
*n*	8,827	6,652	2,175		
Demographic					
Age	76.00 [67.00, 83.50]	76.00 [67.00, 84.00]	74.00 [66.00, 81.00]	<0.001	0.148
Sex				<0.001	0.165
Female	3,481 (39.4)	2,754 (41.4)	727 (33.4)		
Male	5,346 (60.6)	3,898 (58.6)	1,448 (66.6)		
Weight	81.10 [67.82, 96.90]	80.00 [66.80, 96.20]	84.10 [71.50, 98.20]	<0.001	0.093
Race				<0.001	0.184
Asian	230 (2.6)	182 (2.7)	48 (2.2)		
Black	608 (6.9)	523 (7.9)	85 (3.9)		
Hispanic	195 (2.2)	138 (2.1)	57 (2.6)		
Other/Unknown	1,423 (16.1)	1,092 (16.4)	331 (15.2)		
White	6,371 (72.2)	4,717 (70.9)	1,654 (76.0)		
Vital signs					
Temp, °C	98.10 [97.60, 98.70]	98.10 [97.60, 98.80]	98.00 [97.50, 98.60]	<0.001	0.102
HR, beats/min	86.00 [75.00, 102.00]	89.00 [76.00, 105.00]	80.00 [74.00, 90.00]	<0.001	0.381
RR, breaths/min	18.00 [15.00, 23.00]	19.00 [16.00, 24.00]	16.00 [13.00, 20.00]	<0.001	0.545
SBP, mmHg	115.00 [101.00, 132.00]	116.00 [101.00, 134.00]	113.00 [100.00, 126.00]	<0.001	0.191
DBP, mmHg	63.00 [53.00, 75.00]	64.00 [54.00, 77.00]	60.00 [51.00, 69.00]	<0.001	0.284
SpO2, %	98.00 [95.00, 100.00]	98.00 [95.00, 100.00]	100.00 [97.00, 100.00]	<0.001	0.409
Comorbidity, *n* (%)					
VHD	8,204 (92.9)	6,124 (92.1)	2,080 (95.6)	<0.001	0.149
HTN	3,518 (39.9)	2,451 (36.8)	1,067 (49.1)	<0.001	0.249
AKI	4,248 (48.1)	3,568 (53.6)	680 (31.3)	<0.001	0.465
IS	1,035 (11.7)	791 (11.9)	244 (11.2)	0.419	0.021
CKD	2,561 (29.0)	2,031 (30.5)	530 (24.4)	<0.001	0.138
Cancer	1,708 (19.3)	1,309 (19.7)	399 (18.3)	0.182	0.034
HLD	4,136 (46.9)	2,894 (43.5)	1,242 (57.1)	<0.001	0.275
HF	4,317 (48.9)	3,386 (50.9)	931 (42.8)	<0.001	0.163
MI	891 (10.1)	652 (9.8)	239 (11.0)	0.120	0.039
CAD	4,509 (51.1)	3,064 (46.1)	1,445 (66.4)	<0.001	0.420
DM	3,052 (34.6)	2,348 (35.3)	704 (32.4)	0.014	0.062
Severity scores					
SOFA score	6.00 [4.00, 8.00]	6.00 [4.00, 8.00]	5.00 [4.00, 8.00]	<0.001	0.135
GCS score	15.00 [13.00, 15.00]	15.00 [13.00, 15.00]	15.00 [14.00, 15.00]	0.004	0.027
APACHE II score	20.00 [16.00, 25.00]	21.00 [16.00, 26.00]	19.00 [16.00, 24.00]	<0.001	0.162
Biochemistry					
WBC, ×10^−^9/L	11.80 [8.30, 16.40]	11.80 [8.20, 16.60]	11.80 [8.70, 15.85]	0.822	0.072
Hb, g/dL	10.00 [8.60, 11.60]	10.10 [8.60, 11.70]	9.70 [8.50, 11.10]	<0.001	0.189
PLT, ×10^−^9/L	171.00 [124.00, 239.00]	180.00 [128.00, 250.00]	148.00 [114.00, 204.00]	<0.001	0.298
K, mEq/L	4.20 [3.80, 4.70]	4.20 [3.80, 4.70]	4.20 [3.90, 4.60]	0.054	0.017
Na, mEq/L	139.00 [136.00, 141.00]	139.00 [135.00, 142.00]	139.00 [137.00, 141.00]	0.001	0.065
Ca, mg/dL	8.30 [7.80, 8.80]	8.30 [7.80, 8.80]	8.30 [7.90, 8.70]	0.522	0.003
Cr, mg/dL	1.20 [0.80, 1.90]	1.20 [0.90, 2.00]	1.00 [0.80, 1.40]	<0.001	0.302
BUN, mg/dL	25.00 [17.00, 42.00]	28.00 [18.00, 46.00]	19.00 [14.00, 29.00]	<0.001	0.460
PT, s	15.60 [13.60, 19.30]	15.65 [13.50, 20.10]	15.60 [13.90, 17.80]	0.008	0.194
INR	1.40 [1.20, 1.80]	1.40 [1.20, 1.80]	1.40 [1.20, 1.60]	<0.001	0.184
pH	7.38 [7.31, 7.43]	7.37 [7.30, 7.43]	7.39 [7.34, 7.44]	<0.001	0.274
PaO2, mmHg	106.00 [55.00, 256.00]	87.00 [48.00, 170.00]	275.00 [117.00, 360.00]	<0.001	0.951
Lac, mmol/L	1.80 [1.30, 2.70]	1.80 [1.30, 2.70]	2.00 [1.40, 2.80]	<0.001	0.014
Glu, mg/dL	129.00 [107.00, 164.00]	132.00 [107.00, 169.00]	122.00 [105.00, 147.50]	<0.001	0.213
Treatment, *n* (%)					
CRRT	822 (9.3)	671 (10.1)	151 (6.9)	<0.001	0.113
MV	8,042 (91.1)	5,959 (89.6)	2,083 (95.8)	<0.001	0.239
Beta-blockers	558 (6.3%)	389 (5.8%)	169 (7.8%)	0.002	0.08
Warfarin	60 (0.7%)	39 (0.6%)	21 (1.0%)	0.071	0.04
NOACs	229 (2.6%)	190 (2.9%)	39 (1.8%)	0.006	0.07
Outcomes					
Hospital length of stay, days	9.98 [6.33, 17.10]	11.02 [6.90, 18.84]	7.84 [5.40, 12.63]	<0.001	0.316
ICU length of stay, days	3.68 [2.00, 7.18]	3.65 [2.03, 7.10]	3.78 [1.94, 7.50]	0.259	0.080

SMD, standardized mean difference; HR, heart rate; RR, respiratory rate; SBP, systolic blood pressure; DBP, diastolic blood pressure; SpO₂, peripheral oxygen saturation; VHD, valvular heart disease; HTN, hypertension; AKI, acute kidney injury; IS, ischemic stroke; CKD, chronic kidney disease; HLD, hyperlipidemia; HF, heart failure; MI, myocardial infarction; CAD, coronary artery disease; DM, diabetes mellitus; SOFA, sequential organ failure assessment; APACHE II, acute physiology and chronic health evaluation II; GCS, glasgow coma scale; WBC, white blood cell count; Hb, hemoglobin; PLT, platelet count; PT, prothrombin time; INR, international normalized ratio; BUN, blood urea nitrogen; Cr, serum creatinine; Lac, lactate; MV, invasive mechanical ventilation; CRRT, continuous renal replacement therapy; NOACs, non-vitamin K antagonist oral anticoagulants.

In the eICU-CRD validation cohort, 1,955 patients met the study criteria, including 336 early aspirin users and 1,619 non-aspirin users. Overall baseline differences between the two groups were modest, although early aspirin users had a higher prevalence of CAD and MI and were more likely to receive warfarin (Supplementary Table S1).

### 3.2 Clinical outcomes

In the primary cohort, early aspirin exposure was associated with lower 30-day all-cause mortality and lower in-hospital mortality compared with no aspirin exposure within 48 h after ICU admission (Table 1). Total hospital length of stay was shorter in the early aspirin group, whereas ICU length of stay did not differ significantly between groups.

At 30-day follow-up, survivors and non-survivors differed significantly. Aspirin exposure was more frequent among survivors than among non-survivors (28.36% vs. 12.95%) (Supplementary Table S2). In the MIMIC-IV cohort, survivors were younger and had lower heart rate, respiratory rate, lactate, creatinine, and glucose levels, as well as higher PaO₂ values. Survivors also had lower CRRT use but higher use of warfarin and beta-blockers. Non-survivors had higher rates of VHD, AKI, CKD, MI, HF, and CAD, and a lower prevalence of HTN (Supplementary Table S2).

In the eICU-CRD cohort, shorter ICU and hospital lengths of stay were also observed among early aspirin users (Supplementary Table S1). The direction of mortality differences was generally consistent with that observed in the primary cohort.

### 3.3 Kaplan–Meier analysis

**Figure 2:**
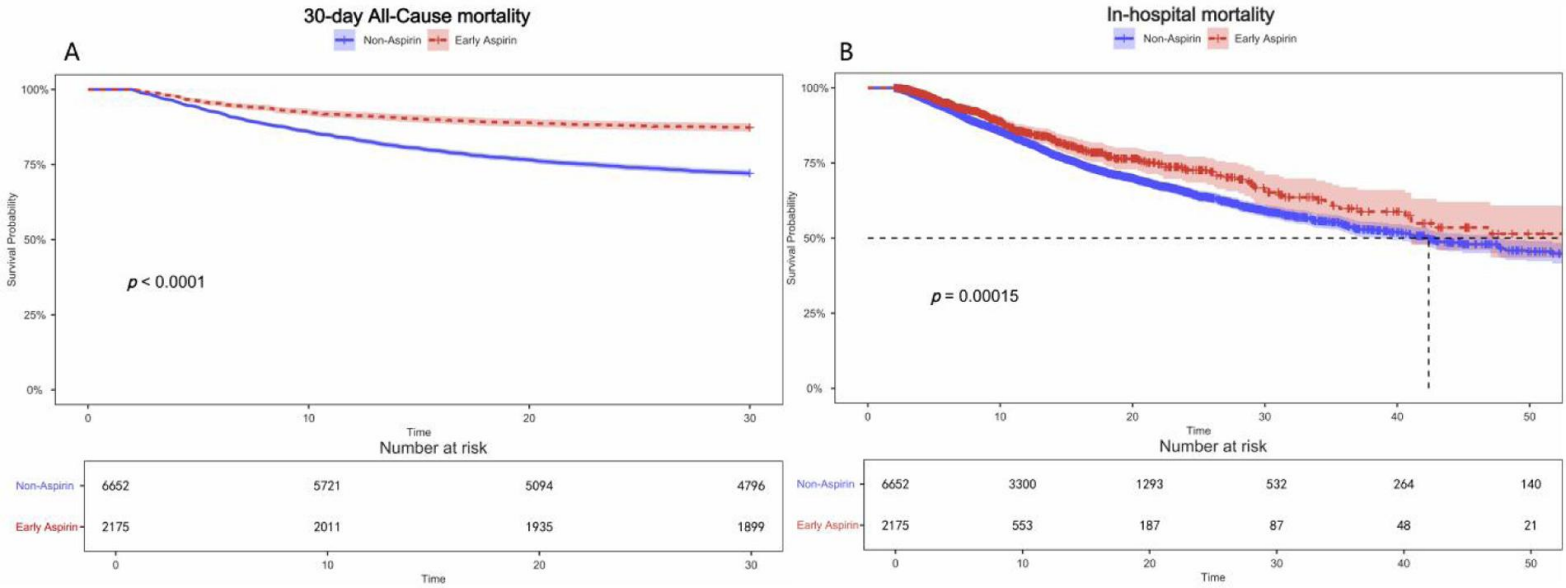
Kaplan–Meier analyses of early aspirin exposure for 30-day all-cause mortality and in-hospital mortality in the MIMIC-IV cohort. **(A)** 30-day all-cause mortality; **(B)** in-hospital mortality. Non-aspirin: no aspirin exposure within 48 h after ICU admission; early aspirin: aspirin exposure before ICU admission or within 48 h after ICU admission.

Kaplan–Meier analysis demonstrated lower cumulative mortality in the early aspirin group than in the non-aspirin group for both 30-day all-cause mortality and in-hospital mortality in the MIMIC-IV cohort, with more pronounced separation for 30-day all-cause mortality (Figure 2).

In the eICU-CRD cohort, Kaplan–Meier analysis also showed lower in-hospital mortality in the early aspirin group (Supplementary Figure S1).

### 3.4 Multivariable Cox regression and overlap-weighted analysis

In the primary cohort, conventional Cox regression models revealed a robust, inverse association between early aspirin exposure and 30-day all-cause mortality. The survival benefit remained significant across sequential adjustments: Model 1 (HR = 0.420, 95% CI: 0.370–0.476, *P* < 0.001), Model 2 (HR = 0.582, 95% CI: 0.511–0.663, *P* < 0.001), and Model 3 (HR = 0.624, 95% CI: 0.547–0.712, *P* < 0.001). Conversely, the protective effect against in-hospital mortality was attenuated after multivariable adjustment, with corresponding HRs of 0.766 (95% CI: 0.667–0.880) in Model 1 and 0.867 (95% CI: 0.753–0.999) in Model 2, ultimately losing statistical significance in Model 3 (HR = 0.890, 95% CI: 0.771–1.026) (Table 2).

**Table 2:** Association between early aspirin exposure and 30-day all-cause mortality and in-hospital mortality in the MIMIC-IV cohort.

Variables	30-day All-Cause mortality			In-hospital mortality		
	HR	95% CI	*P*	HR	95% CI	*P*
Model 1						
Non-aspirin group	1	Ref		1	Ref	
Early Aspirin group	0.42	0.370–0.476	<0.001	0.766	0.667–0.880	<0.001
Model 2						
Non-aspirin group	1	Ref		1	Ref	
Early Aspirin group	0.582	0.511–0.663	<0.001	0.867	0.753–0.999	0.048
Model 3						
Non-aspirin group	1	Ref		1	Ref	
Early Aspirin group	0.624	0.547–0.712	<0.001	0.89	0.771–1.026	0.108

HR, hazard ratio; CI, confidence interval; RR, respiratory rate; SBP, systolic blood pressure; DBP, diastolic blood pressure; HTN, hypertension; IS, ischemic stroke; CKD, chronic kidney disease; DM, diabetes mellitus; HLD, hyperlipidemia; HF, heart failure; MI, myocardial infarction; CAD, coronary artery disease; SOFA, Sequential Organ Failure Assessment; WBC, white blood cell count; Hb, hemoglobin; PLT, platelet count; Glu, glucose; INR, international normalized ratio; BUN, blood urea nitrogen; Cr, serum creatinine; Lac, lactate; CRRT, continuous renal replacement therapy; MV, invasive mechanical ventilation. Non-aspirin: no aspirin exposure within 48 h after ICU admission. Early aspirin: aspirin exposure before ICU admission or within 48 h after ICU admission. Model 1: unadjusted model. Model 2: adjusted for demographic characteristics (age, weight, sex), admission vital signs (HR, RR, SBP, DBP), comorbidities (HTN, IS, CKD, cancer, DM, HLD, HF, MI, CAD), and SOFA score. Model 3: adjusted for variables in Model 2 plus biochemical parameters (WBC, Hb, PLT, Glu, potassium, sodium, INR, BUN, Cr, pH, Lac) and treatment variables (CRRT and MV).

In the overlap-weighted Cox analysis, early aspirin exposure remained associated with lower 30-day all-cause mortality (HR: 0.738, 95% CI: 0.647–0.842; *P* < 0.001), whereas the association with in-hospital mortality was not statistically significant (HR: 1.111, 95% CI: 0.963–1.283; *P* = 0.148) (Table 3).

**Table 3:** Overlap-weighted Cox regression analyses of early aspirin exposure and mortality from the 48-hour landmark after ICU admission in the MIMIC-IV cohort.

Outcome	Method	Comparison	HR (95% CI)	*P*
In-hospital mortality	Overlap weighting	Early Aspirin group vs. Non-aspirin group	1.111 (0.963–1.283)	0.148
30-day All-Cause mortality	Overlap weighting	Early Aspirin group vs. Non-aspirin group	0.738 (0.647–0.842)	<0.001

HR, hazard ratio; CI, confidence interval. Non-aspirin: no aspirin exposure within 48 h after ICU admission. Early aspirin: aspirin exposure before ICU admission or within 48 h after ICU admission.

In the eICU-CRD cohort, multivariable Cox regression analyses showed that early aspirin exposure was associated with lower in-hospital mortality across all three models, including the fully adjusted Model 3 (HR: 0.732, 95% CI: 0.559–0.959; *P* = 0.024). For 30-day in-hospital mortality, the direction of association was similar, but the estimate was attenuated in the fully adjusted model (HR: 0.780, 95% CI: 0.596–1.022; *P* = 0.072) (Supplementary Table S3).

### 3.5 Sensitivity analysis according to aspirin exposure timing

In sensitivity analyses, early aspirin exposure was further divided into aspirin exposure before ICU admission and new aspirin initiation within 48 h after ICU admission. Baseline characteristics differed across the three exposure groups (Supplementary Table S4). Compared with the non-aspirin group, patients with aspirin exposure before ICU admission were generally older and had a higher burden of cardiovascular comorbidity, whereas patients newly initiated on aspirin within 48 h after ICU admission showed a distinct clinical profile, including younger age and shorter hospital stay.

In the three-category sensitivity analysis, the association with lower mortality was observed mainly among patients newly initiated on aspirin within 48 h after ICU admission. By contrast, patients with aspirin exposure before ICU admission did not show a similarly clear association after full adjustment. For in-hospital mortality, the fully adjusted hazard ratio was 1.209 (95% CI: 0.963–1.517; *P* = 0.103) for aspirin exposure before ICU admission and 0.778 (95% CI: 0.656–0.923; *P* = 0.004) for new aspirin initiation within 48 h, both compared with the non-aspirin group. A similar pattern was observed for 30-day all-cause mortality, with the inverse association driven primarily by patients newly initiated on aspirin within 48 h after ICU admission (Supplementary Table S5).

### 3.6 Subgroup analysis

**Figure 3:**
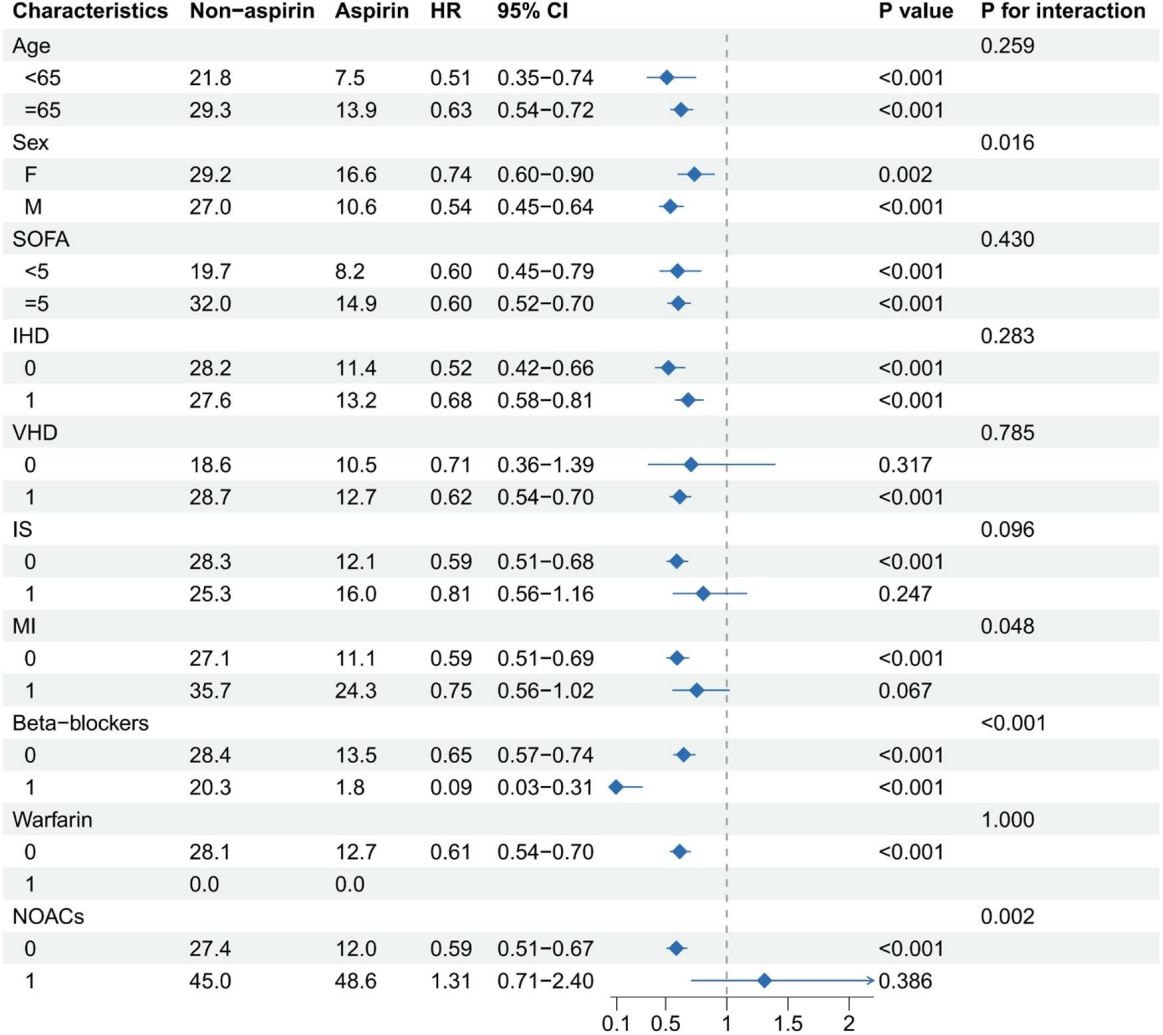
Subgroup analysis of the association between early aspirin exposure and 30-day all-cause mortality in sepsis patients with AF. Non-aspirin: no aspirin exposure within 48 h after ICU admission; early aspirin: aspirin exposure before ICU admission or within 48 h after ICU admission.

In subgroup analyses of 30-day all-cause mortality, early aspirin exposure was associated with lower mortality across most prespecified subgroups (Figure 3). No significant interaction was observed according to age, SOFA score, CAD, VHD, IS, or warfarin use. Significant interactions were identified for sex (P for interaction = 0.016), myocardial infarction (P for interaction = 0.048), beta-blocker use (P for interaction <0.001), and NOAC use (P for interaction = 0.002). The inverse association appeared stronger in male patients and in those receiving beta-blockers, whereas it was attenuated in patients with myocardial infarction and was not evident among NOAC users.

## 4 Discussion

Early aspirin exposure within 48 h after ICU admission was associated with lower 30-day all-cause mortality in sepsis patients with AF. This association remained significant after multivariable adjustment and was further supported by the overlap-weighted analysis. By contrast, the association with in-hospital mortality was less consistent, showing attenuation after full adjustment in the primary cohort and loss of statistical significance in the overlap-weighted analysis. In the external validation cohort, early aspirin exposure remained associated with lower in-hospital mortality, whereas the association with 30-day in-hospital mortality was directionally similar but attenuated in the fully adjusted model. Taken together, these findings suggest that the most consistent finding in the present study is the association between early aspirin exposure and lower short-term mortality, particularly 30-day all-cause mortality.

These findings should be interpreted within the clinical context of sepsis-associated AF, a phenotype that has repeatedly been linked to adverse outcomes. AF is a common cardiovascular complication in sepsis and may arise in response to inflammatory activation, autonomic imbalance, myocardial dysfunction, and pre-existing structural susceptibility (3, 7, 32, 33). Prior studies by Walkey and colleagues demonstrated that new-onset AF during severe sepsis was associated with increased risks of in-hospital stroke and death, and that AF developing during sepsis was also associated with higher long-term risks of recurrent AF, heart failure, ischemic stroke, and death after discharge (5, 6). Subsequent reviews and cohort studies have further reinforced the view that sepsis-associated AF is not a benign arrhythmia, but rather a clinically meaningful marker of worse short- and long-term prognosis (7, 32, 33). These observations provide an important framework for interpreting the present results, as they indicate that patients with sepsis and AF constitute a particularly high-risk population in whom antithrombotic and anti-inflammatory strategies may be especially relevant.

An important finding of the present study is that the overall association was not uniform across all aspirin-exposed patients. In the three-category sensitivity analysis, the inverse association with mortality was observed mainly among patients newly initiated on aspirin within 48 h after ICU admission, whereas patients with aspirin exposure before ICU admission did not show a similarly clear adjusted association. This distinction is clinically meaningful. Patients already receiving aspirin before ICU admission are likely to differ substantially from those in whom aspirin is initiated during early ICU care with respect to comorbidity burden, treatment indication, and underlying cardiovascular risk. In routine AF care, aspirin has become increasingly restricted as a stroke-prevention strategy, with contemporary management emphasizing oral anticoagulation in appropriately selected patients (24–27). However, sepsis-associated AF represents a distinct inflammatory and prothrombotic state, and the present findings suggest that aspirin exposure during the early ICU phase may carry a different prognostic implication from chronic outpatient aspirin use. Accordingly, the binary early-aspirin analysis should not be interpreted as evidence of a homogeneous association across all aspirin-exposed patients.

From a biological perspective, aspirin may plausibly influence outcomes in sepsis complicated by AF through several related pathways. Sepsis is characterized by dysregulated inflammation, endothelial injury, platelet activation, and immunothrombosis (1, 2, 34), whereas AF may further amplify thrombotic vulnerability and haemodynamic instability through inflammation-related atrial remodeling and procoagulant activation (7, 35, 36). Aspirin irreversibly inhibits platelet cyclooxygenase-1, suppresses thromboxane A₂ generation, and may modulate platelet–leukocyte interactions and inflammation-related endothelial dysfunction (8, 20, 37–39). These mechanisms are particularly relevant to sepsis, in which platelet activation contributes not only to thrombosis but also to immune dysregulation, microvascular injury, and organ dysfunction (20, 40, 41). In addition, platelet–neutrophil interactions and neutrophil extracellular trap formation may provide a further mechanistic link between thrombosis and inflammation in this setting (39, 41). These considerations provide a biologically plausible basis for interpreting the observed association, although they were not directly assessed in the present study.

Against this biological background, existing clinical evidence on aspirin or antiplatelet therapy in sepsis has been mixed but generally suggests potential benefit in selected settings. Several observational studies reported lower mortality among septic patients with prior or early antiplatelet exposure (16, 17, 21–23, 42), whereas others found no clear association after adjustment (18, 19). Meta-analyses have also yielded heterogeneous but broadly supportive results. Ouyang et al. found that antiplatelet therapy, particularly aspirin, was associated with reduced mortality in sepsis, and a more recent systematic review reached a similar conclusion while emphasizing the substantial heterogeneity of the available observational evidence (40, 43). Taken together, the available evidence supports a possible prognostic role of aspirin in at least some sepsis populations, while also highlighting substantial heterogeneity across studies.

The subgroup analysis further suggested that the association between early aspirin exposure and lower 30-day mortality may vary across clinical subgroups. Significant interactions were identified for sex, myocardial infarction, beta-blocker use, and NOAC use. The inverse association appeared stronger in male patients and in those receiving beta-blockers, whereas it was attenuated in patients with myocardial infarction and was not evident among NOAC users. These findings should be interpreted cautiously, as subgroup analyses are inherently more vulnerable to instability and may be affected by smaller sample sizes in some strata. Nevertheless, these subgroup findings may still be clinically informative. Given that AF management is fundamentally intertwined with antithrombotic therapy (26), the weaker association among NOAC users may suggest that the prognostic relevance of aspirin differs according to the surrounding antithrombotic treatment context. Similarly, the interaction with myocardial infarction may reflect competing cardiovascular risk profiles or treatment-selection differences rather than true biological effect modification.

## 5 Limitations

This study has several limitations. First, despite the 48-hour exposure framework, time-related bias and confounding by indication cannot be fully excluded in a retrospective observational study. Second, the association was more reproducible for 30-day all-cause mortality than for in-hospital mortality across analytic approaches. Third, the external validation results were supportive but not fully concordant. In addition, exposure classification was based on recorded medication data and may not fully reflect adherence, dosing intent, or the clinical context underlying treatment decisions. The eICU-CRD cohort did not permit replication of long-term post-discharge outcomes. Finally, because the present study was designed to assess association rather than causation, the findings should be regarded as hypothesis-generating and require confirmation in prospective studies.

## 6 Conclusion

Among patients with sepsis and AF, early aspirin exposure within 48 h after ICU admission was associated with lower short-term mortality, with the most consistent signal observed for 30-day all-cause mortality. This association appeared to be driven mainly by patients newly initiated on aspirin within 48 h after ICU admission rather than by those already receiving aspirin before ICU admission. These findings suggest that aspirin may have prognostic relevance in this high-risk population, while prospective studies are needed to clarify causality, identify the patients most likely to benefit, and define the optimal timing of exposure.

## Data Availability

The data used in this study were obtained from the MIMIC-IV (Medical Information Mart for Intensive Care IV) and eICU Collaborative Research Database (eICU-CRD), which are publicly available, de-identified databases. The establishment of these databases was approved by the institutional review boards of the Massachusetts Institute of Technology and participating institutions.
