# Supplementary material for: Aspirin is associated with improved outcomes in sepsis patients with atrial fibrillation: an analysis of the MIMIC-IV and eICU-CRD databases
Source: Front Cardiovasc Med. 2026 May 26;13:1796096. doi: 10.3389/fcvm.2026.1796096 (PMC13246384; doi:10.3389/fcvm.2026.1796096)
Supplement: Supplementary file 1 [file Supplementaryfile1.docx]

**Supplementary Material**

**Supplementary Table S1.** Baseline characteristics of sepsis patients with AF according to early aspirin exposure in the eICU-CRD database

| **Variable** | **Overall** | **Non aspirin** | **Early Aspirin** | **P value** | **SMD** |
| --- | --- | --- | --- | --- | --- |
| n | 1955 | 1619 | 336 |  |  |
| Demographic |  |  |  |  |  |
| Age, years | 75.00 [66.00, 83.00] | 75.00 [66.00, 83.00] | 76.00 [66.00, 84.00] | 0.333 | 0.047 |
| Male | 1076 (55.0) | 900 (55.6) | 176 (52.4) | 0.310 | 0.064 |
| Race |  |  |  | 0.086 | 0.165 |
| White | 1568 (80.2) | 1288 (79.6) | 280 (83.3) |  |  |
| Black | 100 (5.1) | 80 (4.9) | 20 (6.0) |  |  |
| Hispanic | 86 (4.4) | 72 (4.4) | 14 (4.2) |  |  |
| Other/Unknown | 201 (10.3) | 179 (11.1) | 22 (6.5) |  |  |
| Height, cm | 170.00 [162.50, 177.80] | 170.00 [162.50, 177.80] | 167.60 [160.00, 177.80] | 0.260 | 0.058 |
| Weight, kg | 81.50 [66.70, 98.40] | 80.90 [66.70, 98.40] | 83.96 [67.07, 98.85] | 0.385 | 0.040 |
| Vital signs |  |  |  |  |  |
| HR, beats/min | 103.00 [86.00, 120.00] | 103.00 [86.00, 120.00] | 101.00 [86.00, 117.00] | 0.318 | 0.064 |
| RR, breaths/min | 22.00 [19.00, 27.00] | 22.00 [18.00, 27.00] | 23.00 [19.00, 28.00] | 0.115 | 0.101 |
| SBP, (mmHg) | 110.00 [96.00, 129.00] | 110.00 [96.00, 128.50] | 111.50 [97.00, 131.00] | 0.313 | 0.052 |
| DBP, (mmHg) | 62.00 [52.00, 74.00] | 62.00 [52.00, 73.00] | 62.00 [51.75, 76.00] | 0.601 | 0.032 |
| Comorbidity, n (%) |  |  |  |  |  |
| VHD | 120 (6.1) | 93 (5.7) | 27 (8.0) | 0.142 | 0.091 |
| HTN | 441 (22.6) | 374 (23.1) | 67 (19.9) | 0.234 | 0.077 |
| AKI | 1679 (85.9) | 1401 (86.5) | 278 (82.7) | 0.083 | 0.105 |
| IS | 428 (21.9) | 354 (21.9) | 74 (22.0) | 1.000 | 0.004 |
| MI | 257 (13.1) | 193 (11.9) | 64 (19.0) | 0.001 | 0.198 |
| CAD | 367 (18.8) | 266 (16.4) | 101 (30.1) | <0.001 | 0.327 |
| DM | 724 (37.0) | 614 (37.9) | 110 (32.7) | 0.084 | 0.109 |
| Severity scores |  |  |  |  |  |
| SOFA score | 5.00 [3.00, 8.00] | 5.00 [3.00, 8.00] | 5.00 [3.00, 8.00] | 0.451 | 0.041 |
| APACHE II score | 73.00 [58.00, 89.00] | 74.00 [59.00, 89.00] | 71.00 [57.75, 85.25] | 0.103 | 0.088 |
| GCS score | 15.00 [12.00, 15.00] | 15.00 [12.00, 15.00] | 15.00 [13.00, 15.00] | 0.001 | 0.141 |
| Biochemistry |  |  |  |  |  |
| WBC, ×10^9/L | 17.10 [12.15, 23.90] | 17.30 [12.30, 23.90] | 16.40 [11.86, 23.83] | 0.321 | 0.0a76 |
| Hb, g/dL | 11.20 [9.90, 12.60] | 11.10 [9.90, 12.50] | 11.50 [9.90, 12.80] | 0.033 | 0.118 |
| PLT, ×10^9/L | 244.00 [173.00, 335.50] | 245.00 [173.50, 337.00] | 241.00 [170.75, 328.25] | 0.807 | 0.015 |
| K, mEq/L | 4.60 [4.20, 5.10] | 4.60 [4.20, 5.20] | 4.50 [4.20, 5.00] | 0.036 | 0.119 |
| Na, mEq/L | 143.00 [139.00, 147.00] | 143.00 [140.00, 147.00] | 142.00 [139.00, 146.00] | 0.006 | 0.163 |
| Cr, mg/dL | 1.69 [1.08, 2.95] | 1.68 [1.08, 2.96] | 1.73 [1.05, 2.89] | 0.931 | 0.025 |
| BUN, mg/dL | 43.00 [27.00, 67.00] | 44.00 [27.00, 69.00] | 40.00 [28.00, 60.25] | 0.061 | 0.112 |
| INR | 1.75 [1.30, 3.10] | 1.80 [1.30, 3.10] | 1.61 [1.27, 3.14] | 0.472 | 0.006 |
| Glu, mg/dL | 211.00 [163.00, 286.00] | 212.00 [162.00, 286.00] | 211.00 [165.00, 289.25] | 0.704 | 0.017 |
| Treatment, n (%) |  |  |  |  |  |
| MV | 594 (30.4) | 497 (30.7) | 97 (28.9) | 0.550 | 0.040 |
| Warfarin | 79 (4.0) | 47 (2.9) | 32 (9.5) | <0.001 | 0.277 |
| Outcomes |  |  |  |  |  |
| Hospital length of stay, days | 9.74 [5.97, 16.18] | 9.89 [6.13, 16.84] | 8.90 [5.57, 14.16] | 0.004 | 0.189 |
| ICU length of stay, days | 4.00 [2.54, 7.92] | 4.04 [2.54, 8.12] | 3.81 [2.49, 6.88] | 0.039 | 0.170 |

**Abbreviations:** SMD, standardized mean difference; HR, heart rate; RR, respiratory rate; SBP, systolic blood pressure; DBP, diastolic blood pressure; AKI, acute kidney injury; VHD, valvular heart disease; IS, ischemic stroke; DM, diabetes mellitus; HTN, hypertension; CAD, coronary artery disease; MI, myocardial infarction; SOFA, Sequential Organ Failure Assessment; APACHE II, Acute Physiology and Chronic Health Evaluation II; GCS, Glasgow Coma Scale; WBC, white blood cell count; Hb, hemoglobin; PLT, platelet count; K, potassium; Na, sodium; Glu, glucose; INR, international normalized ratio; BUN, blood urea nitrogen; Cr, serum creatinine; MV, invasive mechanical ventilation.

**Supplementary Table S2.** Baseline characteristics of sepsis patients with AF in the MIMIC-IV database according to 30-day all-cause mortality status

| **30-day all-cause mortality in MIMIC-IV** | | | | | |
| --- | --- | --- | --- | --- | --- |
| **Variable** | **Overall** | **Alive** | **Death** | **P value** | **SMD** |
| **N** | 8827 | 6695 | 2132 |  |  |
| **Demographic** |  |  |  |  |  |
| age | 76.00 [67.00, 83.50] | 75.00 [66.00, 83.00] | 79.00 [70.00, 86.00] | <0.001 | 0.308 |
| sex |  |  |  | <0.001 | 0.112 |
| Female | 3481 (39.4) | 2551 (38.1) | 930 (43.6) |  |  |
| Male | 5346 (60.6) | 4144 (61.9) | 1202 (56.4) |  |  |
| weight | 81.10 [67.82, 96.90] | 82.70 [69.10, 98.12] | 76.25 [63.68, 92.30] | <0.001 | 0.227 |
| race |  |  |  | <0.001 | 0.151 |
| Asian | 230 (2.6) | 169 (2.5) | 61 (2.9) |  |  |
| Black | 608 (6.9) | 457 (6.8) | 151 (7.1) |  |  |
| Hispanic | 195 (2.2) | 150 (2.2) | 45 (2.1) |  |  |
| Other/Unknown | 1423 (16.1) | 991 (14.8) | 432 (20.3) |  |  |
| White | 6371 (72.2) | 4928 (73.6) | 1443 (67.7) |  |  |
| **Vital signs** |  |  |  |  |  |
| Temp, °C | 98.10 [97.60, 98.70] | 98.10 [97.60, 98.70] | 98.00 [97.50, 98.70] | <0.001 | 0.099 |
| HR, beats/min | 86.00 [75.00, 102.00] | 85.00 [75.00, 100.00] | 92.00 [78.00, 108.00] | <0.001 | 0.262 |
| RR, breaths/min | 18.00 [15.00, 23.00] | 18.00 [14.00, 22.00] | 20.00 [17.00, 25.00] | <0.001 | 0.385 |
| SBP, mmHg | 115.00 [101.00, 132.00] | 115.00 [101.00, 132.00] | 115.00 [100.00, 134.00] | 0.685 | 0.013 |
| DBP, mmHg | 63.00 [53.00, 75.00] | 62.00 [53.00, 74.50] | 64.00 [53.00, 78.25] | <0.001 | 0.098 |
| SpO2, % | 98.00 [95.00, 100.00] | 99.00 [95.00, 100.00] | 97.00 [94.00, 100.00] | <0.001 | 0.297 |
| **Comorbidity, n (%)** |  |  |  |  |  |
| VHD | 8204 (92.9) | 6181 (92.3) | 2023 (94.9) | <0.001 | 0.105 |
| HTN | 3518 (39.9) | 2850 (42.6) | 668 (31.3) | <0.001 | 0.234 |
| AKI | 4248 (48.1) | 2818 (42.1) | 1430 (67.1) | <0.001 | 0.518 |
| IS | 1035 (11.7) | 796 (11.9) | 239 (11.2) | 0.418 | 0.021 |
| CKD | 2561 (29.0) | 1815 (27.1) | 746 (35.0) | <0.001 | 0.171 |
| Cancer | 1708 (19.3) | 1246 (18.6) | 462 (21.7) | 0.002 | 0.076 |
| HLD | 4136 (46.9) | 3254 (48.6) | 882 (41.4) | <0.001 | 0.146 |
| HF | 4317 (48.9) | 3127 (46.7) | 1190 (55.8) | <0.001 | 0.183 |
| MI | 891 (10.1) | 602 (9.0) | 289 (13.6) | <0.001 | 0.145 |
| CAD | 4509 (51.1) | 3473 (51.9) | 1036 (48.6) | 0.009 | 0.066 |
| DM | 3052 (34.6) | 2316 (34.6) | 736 (34.5) | 0.973 | 0.002 |
| **Severity scores** |  |  |  |  |  |
| SOFA score | 6.00 [4.00, 8.00] | 5.00 [4.00, 8.00] | 7.00 [5.00, 10.00] | <0.001 | 0.475 |
| GCS score | 15.00 [13.00, 15.00] | 15.00 [13.00, 15.00] | 14.00 [12.00, 15.00] | <0.001 | 0.134 |
| APACHE II score | 20.00 [16.00, 25.00] | 19.00 [16.00, 24.00] | 23.00 [19.00, 28.00] | <0.001 | 0.539 |
| **Biochemistry** |  |  |  |  |  |
| WBC, ×10^9/L | 11.80 [8.30, 16.40] | 11.60 [8.30, 16.00] | 12.30 [8.60, 17.70] | <0.001 | 0.146 |
| Hb, g/dL | 10.00 [8.60, 11.60] | 10.00 [8.70, 11.60] | 10.00 [8.50, 11.62] | 0.324 | 0.015 |
| PLT, ×10^9/L | 171.00 [124.00, 239.00] | 168.00 [123.00, 233.00] | 184.00 [125.00, 260.25] | <0.001 | 0.119 |
| K, mEq/L | 4.20 [3.80, 4.70] | 4.20 [3.80, 4.60] | 4.20 [3.80, 4.80] | 0.005 | 0.074 |
| Na, mEq/L | 139.00 [136.00, 141.00] | 139.00 [136.00, 141.00] | 138.00 [135.00, 142.00] | 0.002 | 0.050 |
| Ca, mg/dL | 8.30 [7.80, 8.80] | 8.30 [7.90, 8.70] | 8.30 [7.80, 8.80] | 0.849 | 0.012 |
| Cr, mg/dL | 1.20 [0.80, 1.90] | 1.10 [0.80, 1.70] | 1.40 [1.00, 2.40] | <0.001 | 0.250 |
| BUN, mg/dL | 25.00 [17.00, 42.00] | 22.00 [16.00, 37.00] | 35.00 [22.00, 54.25] | <0.001 | 0.463 |
| PT, s | 15.60 [13.60, 19.30] | 15.50 [13.60, 18.55] | 16.30 [13.80, 22.20] | <0.001 | 0.209 |
| INR | 1.40 [1.20, 1.80] | 1.40 [1.20, 1.70] | 1.50 [1.20, 2.10] | <0.001 | 0.207 |
| pH | 7.38 [7.31, 7.43] | 7.38 [7.32, 7.43] | 7.36 [7.28, 7.42] | <0.001 | 0.246 |
| PaO2, mmHg | 106.00 [55.00, 256.00] | 124.00 [63.00, 296.00] | 76.00 [44.00, 130.00] | <0.001 | 0.613 |
| Lac, mmol/L | 1.80 [1.30, 2.70] | 1.80 [1.30, 2.70] | 1.90 [1.40, 3.00] | <0.001 | 0.222 |
| Glu, mg/dL | 129.00 [107.00, 164.00] | 127.00 [106.00, 159.00] | 136.00 [107.00, 179.00] | <0.001 | 0.160 |
| **Treatment, n (%)** |  |  |  |  |  |
| CRRT | 822 (9.3) | 424 (6.3) | 398 (18.7) | <0.001 | 0.380 |
| MV | 8042 (91.1) | 6095 (91.0) | 1947 (91.3) | 0.720 | 0.010 |
| Aspirin | 2,175.00 (24.64%) | 1,899.00 (28.36%) | 276.00 (12.95%) | <0.001 | 0.39 |
| Beta-blockers | 558.00 (6.32%) | 476.00 (7.11%) | 82.00 (3.85%) | <0.001 | 0.14 |
| Warfarin | 60.00 (0.68%) | 60.00 (0.90%) | 0.00 (0.00%) | <0.001 | 0.13 |
| NOACs | 229.00 (2.59%) | 124.00 (1.85%) | 105.00 (4.92%) | <0.001 | 0.17 |
| **Survival time** |  |  |  |  |  |
| Hospital length of stay, days | 9.98 [6.33, 17.10] | 10.06 [6.48, 17.89] | 9.74 [5.86, 15.71] | <0.001 | 0.257 |
| ICU length of stay, days | 3.68 [2.00, 7.18] | 3.68 [2.01, 7.26] | 3.67 [2.00, 6.89] | 0.156 | 0.070 |

**Abbreviations:** SMD, standardized mean difference; HR, heart rate; RR, respiratory rate; SBP, systolic blood pressure; DBP, diastolic blood pressure; SpO₂, peripheral oxygen saturation; VHD, valvular heart disease; HTN, hypertension; AKI, acute kidney injury; IS, ischemic stroke; CKD, chronic kidney disease; HLD, hyperlipidemia; HF, heart failure; MI, myocardial infarction; CAD, coronary artery disease; DM, diabetes mellitus; SOFA, Sequential Organ Failure Assessment; APACHE II, Acute Physiology and Chronic Health Evaluation II; GCS, Glasgow Coma Scale; WBC, white blood cell count; Hb, hemoglobin; PLT, platelet count; K, potassium; Na, sodium; Ca, total calcium; PT, prothrombin time; INR, international normalized ratio; BUN, blood urea nitrogen; Cr, serum creatinine; Lac, lactate; Glu, glucose; CRRT, continuous renal replacement therapy; MV, invasive mechanical ventilation; NOACs, non-vitamin K antagonist oral anticoagulants.

**Supplementary Table S3.** Association between early aspirin exposure and mortality in sepsis patients with AF: multivariable-adjusted Cox proportional hazards models in the eICU-CRD database

|  | **Model1** |  | **Model 2** |  | **Model 3** |  |
| --- | --- | --- | --- | --- | --- | --- |
| **Primary outcomes** | **HR (95%CI)** | **p** | **HR (95%CI)** | **p** | **HR (95%CI)** | **p** |
| In-hospital mortality |  |  |  |  |  |  |
| Non-aspirin group | Reference |  | Reference |  | Reference |  |
| Early Aspirin  Group | 0.742 (0.570–0.966) | 0.026 | 0.722 (0.553–0.943) | 0.017 | 0.732 (0.559–0.959) | 0.024 |
| 30-day in-hospital mortality |  |  |  |  |  |  |
| Non-aspirin group | Reference |  | Reference |  | Reference |  |
| Early Aspirin  group | 0.777 (0.596–1.011) | 0.061 | 0.760 (0.582–0.992) | 0.044 | 0.780 (0.596–1.022) | 0.072 |

**Abbreviations:** HR, hazard ratio; CI, confidence interval; AKI, acute kidney injury; VHD, valvular heart disease; IS, ischemic stroke; DM, diabetes mellitus; HTN, hypertension; CAD, coronary artery disease; MI, myocardial infarction; SOFA, Sequential Organ Failure Assessment; WBC, white blood cell count; Hb, hemoglobin; PLT, platelet count; Glu, glucose; Lac, lactate; PT, prothrombin time; INR, international normalized ratio; BUN, blood urea nitrogen; Cr, serum creatinine; MV, invasive mechanical ventilation.
Non-aspirin: no aspirin exposure within 48 h after ICU admission. Early aspirin: aspirin exposure before ICU admission or within 48 h after ICU admission.
Model 1: unadjusted model. Model 2: adjusted for demographic characteristics (age, weight, sex), admission vital signs (HR, RR, SBP, DBP), comorbidities (AKI, VHD, IS, DM, HTN, CAD, MI), SOFA score, biochemical parameters (WBC, Hb, PLT, Glu, potassium, sodium, Lac, PT, Cr), treatment (MV), and medication (warfarin). Model 3: adjusted for variables in Model 2 plus biochemical parameters (WBC, Hb, PLT, Glu, potassium, sodium, INR, BUN, Cr) and treatment (MV).

**Supplementary Table S4.** Baseline characteristics of sepsis patients with AF, stratified by aspirin exposure status and timing in the MIMIC-IV database

| **Variable** | **Overall** | **Non aspirin by 48 h** | **Aspirin before ICU admission** | **New aspirin within 48 h** | **P value** |
| --- | --- | --- | --- | --- | --- |
| **n** | 8827 | 6652 | 383 | 1792 |  |
| **Demographic** |  |  |  |  |  |
| age | 76.00 [67.00, 83.50] | 76.00 [67.00, 84.00] | 79.00 [70.00, 86.00] | 73.00 [66.00, 80.00] | <0.001 |
| sex |  |  |  |  | <0.001 |
| Female | 3481 (39.4) | 2754 (41.4) | 156 (40.7) | 571 (31.9) |  |
| Male | 5346 (60.6) | 3898 (58.6) | 227 (59.3) | 1221 (68.1) |  |
| weight | 81.00 [67.80, 96.76] | 80.00 [66.80, 96.10] | 78.22 [67.88, 93.32] | 85.53 [72.50, 98.99] | <0.001 |
| race |  |  |  |  | <0.001 |
| Asian | 230 (2.6) | 182 (2.7) | 4 (1.0) | 44 (2.5) |  |
| Black | 608 (6.9) | 523 (7.9) | 18 (4.7) | 67 (3.7) |  |
| Hispanic | 195 (2.2) | 138 (2.1) | 8 (2.1) | 49 (2.7) |  |
| Other/Unknown | 1423 (16.1) | 1092 (16.4) | 42 (11.0) | 289 (16.1) |  |
| White | 6371 (72.2) | 4717 (70.9) | 311 (81.2) | 1343 (74.9) |  |
| **Vital signs** |  |  |  |  |  |
| Temp, °C | 98.10 [97.60, 98.70] | 98.10 [97.60, 98.80] | 98.00 [97.40, 98.60] | 98.00 [97.60, 98.60] | <0.001 |
| HR, beats/min | 86.00 [75.00, 102.00] | 89.00 [76.00, 105.00] | 84.00 [73.00, 101.00] | 80.00 [74.00, 88.00] | <0.001 |
| RR, breaths/min | 18.00 [15.00, 23.00] | 19.00 [16.00, 24.00] | 18.00 [14.00, 23.00] | 15.00 [13.00, 18.00] | <0.001 |
| SBP, mmHg | 115.00 [101.00, 132.00] | 116.00 [101.00, 134.00] | 112.00 [99.00, 129.00] | 113.00 [101.00, 125.00] | <0.001 |
| DBP, mmHg | 63.00 [53.00, 75.00] | 64.00 [54.00, 77.00] | 59.00 [50.00, 70.00] | 60.00 [52.00, 69.00] | <0.001 |
| SpO2, % | 98.00 [95.00, 100.00] | 98.00 [95.00, 100.00] | 98.00 [95.00, 100.00] | 100.00 [98.00, 100.00] | <0.001 |
| **Comorbidity, n (%)** |  |  |  |  |  |
| VHD | 8204 (92.9) | 6124 (92.1) | 364 (95.0) | 1716 (95.8) | <0.001 |
| HTN | 3518 (39.9) | 2451 (36.8) | 162 (42.3) | 905 (50.5) | <0.001 |
| AKI | 4248 (48.1) | 3568 (53.6) | 176 (46.0) | 504 (28.1) | <0.001 |
| IS | 1035 (11.7) | 791 (11.9) | 55 (14.4) | 189 (10.5) | 0.076 |
| CKD | 2561 (29.0) | 2031 (30.5) | 123 (32.1) | 407 (22.7) | <0.001 |
| Cancer | 1708 (19.3) | 1309 (19.7) | 63 (16.4) | 336 (18.8) | 0.232 |
| HLD | 4136 (46.9) | 2894 (43.5) | 178 (46.5) | 1064 (59.4) | <0.001 |
| HF | 4317 (48.9) | 3386 (50.9) | 234 (61.1) | 697 (38.9) | <0.001 |
| MI | 891 (10.1) | 652 (9.8) | 33 (8.6) | 206 (11.5) | 0.066 |
| CAD | 4509 (51.1) | 3064 (46.1) | 250 (65.3) | 1195 (66.7) | <0.001 |
| DM | 3052 (34.6) | 2348 (35.3) | 138 (36.0) | 566 (31.6) | 0.011 |
| **Severity scores** |  |  |  |  |  |
| SOFA score | 6.00 [4.00, 8.00] | 6.00 [4.00, 8.00] | 6.00 [4.00, 8.00] | 5.00 [4.00, 8.00] | 0.001 |
| GCS score | 15.00 [13.00, 15.00] | 15.00 [13.00, 15.00] | 15.00 [13.00, 15.00] | 15.00 [14.00, 15.00] | 0.006 |
| APACHE II score | 20.00 [16.00, 25.00] | 21.00 [16.00, 26.00] | 21.00 [17.00, 25.00] | 19.00 [16.00, 24.00] | <0.001 |
| **Biochemistry** |  |  |  |  |  |
| WBC, ×10^9/L | 11.80 [8.30, 16.40] | 11.80 [8.20, 16.60] | 11.70 [7.80, 15.85] | 11.90 [8.80, 15.83] | 0.271 |
| Hb, g/dL | 10.00 [8.60, 11.60] | 10.10 [8.60, 11.70] | 9.80 [8.70, 11.25] | 9.70 [8.50, 11.00] | <0.001 |
| PLT, ×10^9/L | 171.00 [124.00, 239.00] | 180.00 [128.00, 250.00] | 178.00 [123.00, 243.00] | 145.00 [113.00, 197.00] | <0.001 |
| K, mEq/L | 4.20 [3.80, 4.70] | 4.20 [3.80, 4.70] | 4.20 [3.80, 4.60] | 4.20 [3.90, 4.60] | 0.041 |
| Na, mEq/L | 139.00 [136.00, 141.00] | 139.00 [135.00, 142.00] | 139.00 [135.00, 141.00] | 139.00 [137.00, 141.00] | 0.001 |
| Ca, mg/dL | 8.30 [7.80, 8.80] | 8.30 [7.80, 8.80] | 8.30 [7.90, 8.70] | 8.30 [7.90, 8.60] | 0.666 |
| Cr, mg/dL | 1.20 [0.80, 1.90] | 1.20 [0.90, 2.00] | 1.20 [0.90, 1.90] | 0.90 [0.70, 1.30] | <0.001 |
| BUN, mg/dL | 25.00 [17.00, 42.00] | 28.00 [18.00, 46.00] | 27.00 [18.00, 43.00] | 18.00 [14.00, 26.00] | <0.001 |
| PT, s | 15.60 [13.60, 19.30] | 15.65 [13.50, 20.10] | 15.80 [13.45, 19.65] | 15.55 [14.00, 17.60] | 0.010 |
| INR | 1.40 [1.20, 1.80] | 1.40 [1.20, 1.80] | 1.40 [1.20, 1.80] | 1.40 [1.30, 1.60] | <0.001 |
| pH | 7.38 [7.31, 7.43] | 7.37 [7.30, 7.43] | 7.38 [7.32, 7.43] | 7.40 [7.35, 7.44] | <0.001 |
| PaO2, mmHg | 106.00 [55.00, 256.00] | 87.00 [48.00, 170.00] | 135.00 [72.50, 305.00] | 291.00 [146.75, 365.00] | <0.001 |
| Lac, mmol/L | 1.80 [1.30, 2.70] | 1.80 [1.30, 2.70] | 1.80 [1.20, 2.75] | 2.00 [1.40, 2.80] | <0.001 |
| Glu, mg/dL | 129.00 [107.00, 164.00] | 132.00 [107.00, 169.00] | 126.00 [106.00, 162.50] | 122.00 [105.00, 145.00] | <0.001 |
| **Treatment, n (%)** |  |  |  |  |  |
| CRRT | 822 (9.3) | 671 (10.1) | 42 (11.0) | 109 (6.1) | <0.001 |
| MV | 8042 (91.1) | 5959 (89.6) | 353 (92.2) | 1730 (96.5) | <0.001 |
| Beta-blockers | 558 (6.3) | 389 (5.8) | 30 (7.8) | 139 (7.8) | 0.006 |
| Warfarin | 60 (0.7) | 39 (0.6) | 8 (2.1) | 13 (0.7) | 0.002 |
| NOACs | 229 (2.6) | 190 (2.9) | 14 (3.7) | 25 (1.4) | 0.001 |
| **Outcomes** |  |  |  |  |  |
| Hospital length of stay, days | 9.98 [6.33, 17.10] | 11.02 [6.90, 18.84] | 9.72 [6.27, 17.06] | 7.40 [5.32, 11.69] | <0.001 |
| ICU length of stay, days | 3.68 [2.00, 7.18] | 3.65 [2.03, 7.10] | 4.07 [1.94, 8.16] | 3.68 [1.93, 7.37] | 0.158 |

**Abbreviations:** VHD, valvular heart disease; HTN, hypertension; AKI, acute kidney injury; IS, ischemic stroke; CKD, chronic kidney disease; HLD, hyperlipidemia; HF, heart failure; MI, myocardial infarction; CAD, coronary artery disease; DM, diabetes mellitus; SOFA, Sequential Organ Failure Assessment; APACHE II, Acute Physiology and Chronic Health Evaluation II; GCS, Glasgow Coma Scale; WBC, white blood cell count; Hb, hemoglobin; PLT, platelet count; K, potassium; Na, sodium; Ca, total calcium; PT, prothrombin time; INR, international normalized ratio; BUN, blood urea nitrogen; Cr, serum creatinine; Lac, lactate; Glu, glucose; CRRT, continuous renal replacement therapy; MV, invasive mechanical ventilation; NOACs, non-vitamin K antagonist oral anticoagulants.
Non-aspirin: no aspirin exposure within 48 h after ICU admission.
Aspirin before ICU admission: aspirin exposure before ICU admission.
New aspirin within 48 h: aspirin exposure initiated within 48 h after ICU admission.

**Supplementary Table S5**. Association between aspirin exposure patterns and mortality in sepsis patients with AF: multivariable-adjusted Cox proportional hazards models in the MIMIC-IV database

| **Outcome** | **Comparison** | **Model 1 HR**  **(95% CI)** | **P** | **Model 2 HR**  **(95% CI)** | **P** | **Model 3 HR**  **(95% CI)** | **P** |
| --- | --- | --- | --- | --- | --- | --- | --- |
| 30-day all-cause mortality | Aspirin before ICU admission vs Non-aspirin | 0.845 (0.685–1.043) | 0.117 | 0.912 (0.738–1.126) | 0.392 | 0.904 (0.731–1.117) | 0.35 |
| 30-day all-cause mortality | New aspirin within 48 h vs Non-aspirin | 0.337 (0.289–0.391) | <0.001 | 0.49 (0.419–0.572) | <0.001 | 0.536 (0.458–0.628) | <0.001 |
| hospital mortality | Aspirin before ICU admission vs Non-aspirin | 1.247 (0.996–1.563) | 0.054 | 1.224 (0.975–1.535) | 0.081 | 1.209 (0.963–1.517) | 0.103 |
| hospital mortality | New aspirin within 48 h vs Non-aspirin | 0.639 (0.541–0.754) | <0.001 | 0.751 (0.634–0.889) | <0.001 | 0.778 (0.656–0.923) | 0.004 |

**Abbreviations:** HR, hazard ratio; CI, confidence interval; HTN, hypertension; IS, ischemic stroke; CKD, chronic kidney disease; DM, diabetes mellitus; HLD, hyperlipidemia; HF, heart failure; MI, myocardial infarction; CAD, coronary artery disease; SOFA, Sequential Organ Failure Assessment; WBC, white blood cell count; Hb, hemoglobin; PLT, platelet count; Glu, glucose; INR, international normalized ratio; BUN, blood urea nitrogen; Cr, serum creatinine; Lac, lactate; CRRT, continuous renal replacement therapy; MV, invasive mechanical ventilation.
Non-aspirin: no aspirin exposure within 48 h after ICU admission.
Aspirin before ICU admission: aspirin exposure before ICU admission.
New aspirin within 48 h: aspirin exposure initiated within 48 h after ICU admission.
Model 1: unadjusted model. Model 2: adjusted for demographic characteristics (age, weight, sex), admission vital signs (HR, RR, SBP, DBP), comorbidities (HTN, IS, CKD, cancer, DM, HLD, HF, MI, CAD), and SOFA score. Model 3: adjusted for variables in Model 2 plus biochemical parameters (WBC, Hb, PLT, Glu, potassium, sodium, INR, BUN, Cr, pH, Lac) and treatment variables (CRRT and MV).

**Supplementary Table S6.** Missingness of baseline variables in the MIMIC-IV database

| **Domain** | **Variable** | **Missing (%)** |
| --- | --- | --- |
| Demographics | Weight | 0.8 |
| Vital signs | Temp (°F) | 1.5 |
|  | SBP | 0.9 |
|  | DBP | 0.9 |
| Severity scores | GCS | 0.1 |
| Laboratory variables | HB | 0.3 |
|  | PLT | 0.5 |
|  | K | 0.1 |
|  | Na | 0.1 |
|  | Ca | 3.0 |
|  | Cr | 0.1 |
|  | BUN | 0.1 |
|  | PT | 2.4 |
|  | INR | 2.4 |
|  | PaO2 | 13.0 |
|  | Lac | 12.7 |

**Abbreviations:** GCS, Glasgow Coma Scale; INR, international normalized ratio; PT, prothrombin time; APTT, activated partial thromboplastin time.

**Supplementary Table S7.** Missingness of baseline variables in the eICU-CRD database

| **Domain** | **Variable** | **Missing, %** |
| --- | --- | --- |
| Demographic | Age | 4.6 |
|  | Sex | 4.6 |
|  | Race | 5.4 |
|  | Height | 5.1 |
|  | Weight | 7.3 |
| Vital signs | HR | 19.3 |
|  | RR | 28.5 |
|  | SBP | 28.5 |
|  | DBP | 28.5 |
| Severity scores | APACHE II score | 16.0 |
|  | GCS score | 27.8 |
| Biochemistry | WBC | 0.5 |
|  | Hb | 0.7 |
|  | PLT | 0.9 |
|  | K | 0.6 |
|  | Na | 0.3 |
|  | Ca | 1.1 |
|  | Cr | 0.4 |
|  | BUN | 0.3 |
|  | INR | 28.4 |
|  | Glu | 22.8 |

**Abbreviations:** GCS, Glasgow Coma Scale; APACHE II, Acute Physiology and Chronic Health Evaluation II; INR, international normalized ratio; BUN, blood urea nitrogen.


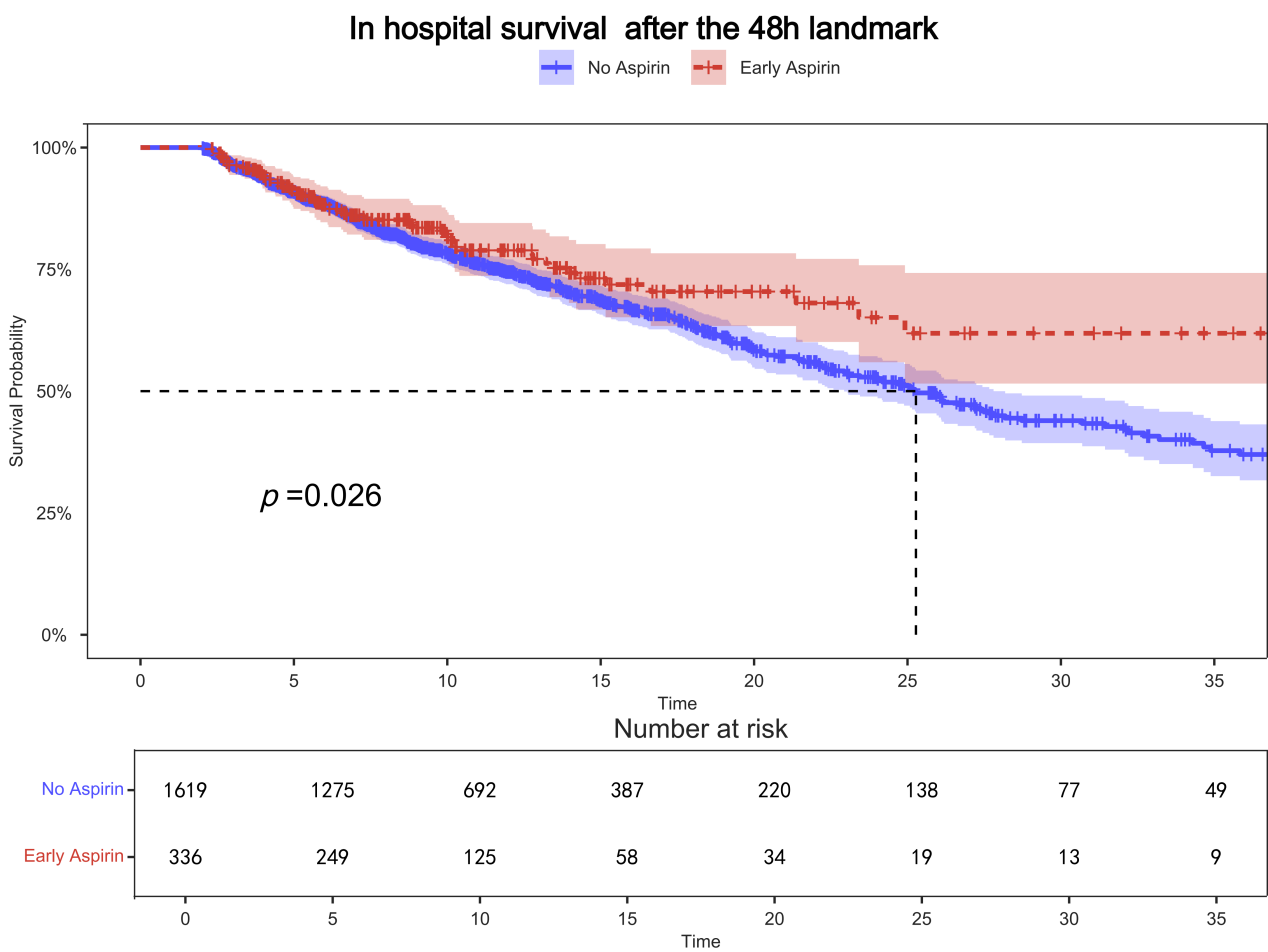


**Supplementary Figure S1.** Kaplan–Meier analysis of early aspirin exposure and in-hospital mortality in the eICU-CRD cohort.
Non-aspirin: no aspirin exposure within 48 h after ICU admission; early aspirin: aspirin exposure before ICU admission or within 48 h after ICU admission.
